# Insights into Disease Progression of Translational Preclinical Rat Model of Interstitial Pulmonary Fibrosis through Endpoint Analysis

**DOI:** 10.3390/cells13060515

**Published:** 2024-03-15

**Authors:** Anil H. Kadam, Jan E. Schnitzer

**Affiliations:** Proteogenomics Research Institute for Systems Medicine (PRISM), 505 Coast Blvd. South, La Jolla, CA 92037, USA; kadamanilh@gmail.com

**Keywords:** rat bleomycin model, disease progression, soluble profibrotic mediators, clinically relevant protein targets, pharmacological interventions, mechanistic studies

## Abstract

Idiopathic pulmonary fibrosis (IPF) is a devastating interstitial lung disease characterized by the relentless deposition of extracellular matrix (ECM), causing lung distortions and dysfunction. Animal models of human IPF can provide great insight into the mechanistic pathways underlying disease progression and a means for evaluating novel therapeutic approaches. In this study, we describe the effect of bleomycin concentration on disease progression in the classical rat bleomycin model. In a dose–response study (1.5, 2, 2.5 U/kg i.t), we characterized lung fibrosis at day 14 after bleomycin challenge using endpoints including clinical signs, inflammatory cell infiltration, collagen content, and bronchoalveolar lavage fluid-soluble profibrotic mediators. Furthermore, we investigated fibrotic disease progression after 2 U/kg i.t. bleomycin administration at days 3, 7, and 14 by quantifying the expression of clinically relevant signaling molecules and pathways, epithelial mesenchymal transition (EMT) biomarkers, ECM components, and histopathology of the lung. A single bleomycin challenge resulted in a progressive fibrotic response in rat lung tissue over 14 days based on lung collagen content, histopathological changes, and modified Ashcroft score. The early fibrogenesis phase (days 3 to 7) is associated with an increase in profibrotic mediators including TGFβ1, IL6, TNFα, IL1β, CINC1, WISP1, VEGF, and TIMP1. In the mid and late fibrotic stages, the TGFβ/Smad and PDGF/AKT signaling pathways are involved, and clinically relevant proteins targeting galectin-3, LPA1, transglutaminase-2, and lysyl oxidase 2 are upregulated on days 7 and 14. Between days 7 and 14, the expressions of vimentin and α-SMA proteins increase, which is a sign of EMT activation. We confirmed ECM formation by increased expressions of procollagen-1Aα, procollagen-3Aα, fibronectin, and CTGF in the lung on days 7 and 14. Our data provide insights on a complex network of several soluble mediators, clinically relevant signaling pathways, and target proteins that contribute to drive the progressive fibrotic phenotype from the early to late phase (active) in the rat bleomycin model. The framework of endpoints of our study highlights the translational value for pharmacological interventions and mechanistic studies using this model.

## 1. Introduction

Idiopathic pulmonary fibrosis (IPF) is a devastating interstitial lung disease characterized by the relentless deposition of extracellular matrix (ECM), causing lung distortions and dysfunction [1]. Bleomycin, a chemotherapeutic drug for human malignant tumors [2], induces progressive inflammation and subsequently fibrosis in rodents [3,4]. Persistent injury leads to the development of pulmonary fibrosis, with excessive collagen production and deposition in the lung [5]. The bleomycin model is well characterized, extensively used, provides invaluable insights into the pathophysiology of pulmonary fibrosis, and allows testing of novel therapeutics [4,6]. Bleomycin induces epithelial cell death, followed by an excessive inflammatory response with the release of soluble inflammatory and profibrotic mediators in the early phase, whereas fibroblast activation, ECM deposition, and development of fibrosis at the molecular and histologic levels occur in the late phase [4,7]. Previous studies showed that neutrophils, lymphocytes, and macrophage inflammatory cells in bronchoalveolar lavage fluid (BALF) were affected to varying degrees in the bleomycin model [8]. Bleomycin induces the release of proinflammatory and profibrotic mediators during fibrosis progression in rodents. Bleomycin-induced epithelial–mesenchymal transition (EMT) is characterized by loss of cell-to-cell contact marked by a decrease in cell adhesion protein E-cadherin and a switch to N-cadherin, a change in morphology from a flattened epithelial cell type into a spindle-shaped, fibroblast-like appearance, and subsequent acquisition of cytoskeletal markers (S100A4, α-SMA, vimentin, β-Catenin), and ECM proteins (procollagen-1Aα, procollagen -3Aα, fibronectin) [9,10,11,12]. Enhanced pathological cross-linking of ECM during fibrosis contributes to increased fibroblast adhesion and proliferation, and enhances the ECM turnover that drives fibrosis pathology [13].

Complicated networks of profibrotic cytokines/growth factors, clinically relevant target molecules, and molecular pathways have been reported in human IPF. Pulmonary fibrosis is initiated and propagated through several distinct signaling pathways or cascades; several of these overlap and converge to activate downstream pathways, which can regulate fibrogenic cellular processes, including mesenchymal proliferation and ECM deposition [14]. Increases in profibrotic cytokines such as transforming growth factor beta-1 (TGFβ1), tumor necrosis factor alpha (TNFα), interleukin 6 (IL6), interleukin 1 beta (IL1β), cytokine-induced neutrophil chemoattractant 1 (CINC1), vascular endothelial growth factor (VEGF), WNT1-inducible-signaling pathway protein 1 (WISP1), and a tissue inhibitor of metalloproteinases 1 (TIMP1) have been reported by various investigators in the bleomycin rodent model. They are implicated in lung fibrosis by their involvement in one or more processes of myofibroblast activation and drive fibrosis pathology in rodents [15,16,17,18,19,20,21,22,23,24,25,26,27,28]. A growing number of preclinical studies have identified promising therapeutic options. Increases in principle pathways/components such as the TGFβ1/Smad pathway, platelet-derived growth factor (PDGF)/AKT pathway, galectin-3, transglutaminase 2, lysophosphatidic acid 1 (LPA1), and lysyl oxidase-like 2 (LOXL2) have been reported in the bleomycin model and human IPF as critical contributors in driving fibrogenesis [27,28,29,30,31,32,33,34,35,36]. 

In our previous studies, we showed that TGFβ1, IL1β, TNFα, IL6, CINC1, TIMP1, and WISP1 were induced by bleomycin within the first 3 days after bleomycin injury [28]. We also confirmed that detectable fibrogenesis occurs at the earliest on day 3 after injury based on collagen content, along with increases in the TGF-β/Smad signaling pathway, PDGF/AKT axis pathway, and expressions of galectin-3, vimentin, and fibronectin in lung homogenate. We ultimately demonstrated the therapeutic value of a novel bispecific antibody as an effective lung therapeutic in pneumonitis [27,28].

In this study, we characterize bleomycin-induced lung fibrosis disease progression in rats by studying time-dependent changes of mediators involved in fibrogenesis, clinically relevant target proteins, and molecular pathways implicated in early, mid, and active late stages of lung fibrosis. To systematically characterize disease progression, we investigated the kinetics of fibrosis drivers such as TGFβ1, IL1β, IL6, TNFα, WISP1, CINC1, TIMP1, and VEGF expression, as well as TGFβ1-Smad-dependent pathways, the PDGF/AKT pathway, and galectin-3, LPA1-1, and LOXL2 expression, along with commonly employed readouts such as inflammatory cell invasion, EMT biomarkers, ECM components, and histological lung changes. We comprehensively identified the time-dependent involvement of a set of drivers in three distinct phases of bleomycin-induced fibrosis in rats. The results of this study shed light on the mechanisms of IPF progression through endpoint analysis of experimental fibrosis. Additionally, we provide a framework of robust, reproducible readouts that represent the various facets of fibrogenesis and the fibrotic phenotype. Importantly, this set of readouts has translational value, enabling the meaningful assessment of IPF. The optimized experimental conditions of this study could be used to quantify the effects of therapeutic interventions and possible disease pathways in the future. Overall, our study provides insights into the disease progression of IPF and the potential utility of the bleomycin rat model for the development of future novel antifibrotic therapeutics with translational success. 

## 2. Materials and Methods

### 2.1. Animals 

Animal experiments were approved by the Institutional Animal Care and Use Committee of PRISM (Approval Number 14-01). Female Sprague Dawley rats weighing 200–220 g (Envigo, Indianapolis, IN, USA) were used after 7 days of acclimatization under pathogen-free conditions. Food and water were available ad libitum.

### 2.2. Induction of Lung Fibrosis and Selected Times for Endpoint Analysis

To induce lung fibrosis, we used pharmaceutical-grade bleomycin for injection, USP (Zydus Hospira Oncology Private Ltd., Gujarat, India) that was dissolved in 1X PBS (pH:7.4). Dose–response studies using predetermined endpoints were performed with 1.5, 2, and 2.5 U/kg bleomycin. Time-dependent characterization of lung fibrosis progression was performed using an array of endpoints after bleomycin challenge with 2 U/kg. Briefly, on day 0, rats were anesthetized with inhaled isoflurane (3–5%) and received a single intratracheal (i.t.) instillation of bleomycin (1.5, 2, or 2.5 U/kg) using an 18-gauge needle attached to a 1 mL tuberculin syringe. Control animals received 300 μL of 1X PBS. After instillation, the rats were allowed to recover from anesthesia, kept warm, and returned to their cages with free access to food and water. Animals were sacrificed on day 3, 7, or 14 post-bleomycin or PBS challenge, and fibrosis-relevant endpoints were analyzed. All animals were monitored daily for their health over the entire duration of the study.

### 2.3. BAL and Collection of BAL Fluid

After 3, 7, or 14 days of bleomycin instillation, the animals were euthanized with Euthasol (100–120 mg/kg) by intraperitoneal (i.p.) injection. The trachea was exposed following a small incision to the skin, and BAL was performed 3 times using a plastic cannula with 2 mL of 1X PBS (pH = 7.4). Volumes of individual BAL aspirates were pooled and aseptically processed immediately for total and differential cell counts, as well as biochemical assays. 

### 2.4. Assessment of Pulmonary Inflammatory Cells

To assess the effect of bleomycin on pulmonary inflammation, BALF samples collected at each time point were analyzed for total leukocytes and differential cells counts, as described previously [27,28,37]. Briefly, we mixed equal volumes of BAL fluid (BALF) and Turk’s solution and counted total leukocytes manually using a hemacytometer (Hausser Scientific, Horsham, PA, USA). The remaining fluid was centrifuged at 4000 RPM for 5 min at 4 °C, and aliquots of BALF supernatant were collected aseptically and stored at −80 °C until analysis. Cell pellets were reconstituted in rat serum and stained with Leishman solution on frosted glass slides (Leica Biosystems, Nußloch, Germany). Using a light microscope (BX2, Olympus, Tokyo, Japan) at 100× magnification, 500 cells/slide were counted. The cells were categorized based upon morphology into neutrophils, lymphocytes, eosinophils, or macrophages.

### 2.5. Lung Collection

To assess the effect of bleomycin on biochemical markers and histology, lung samples were collected at each time point. Briefly, after BAL, right lungs were harvested from the animals for biochemical assays, washed in 1X PBS, placed in 1 mL of PBS containing 0.1% (*v*/*v*) protease and phosphatase inhibitor cocktail, and stored at −80 °C until use. For histology, the left lungs were carefully removed and stored in 10% neutral buffered formalin (NBF).

### 2.6. Assessment of Lung Collagen Content by Sircol Soluble Collagen Assay

Fibrosis was assessed by quantifying total soluble collagen using the Sircol collagen assay kit (Biocolor Ltd., Carrickfergus, UK), as described previously [28]. Briefly, the wet right lungs were washed in 1X PBS and homogenized in 5 mL of CHAPS detergent buffer. The lung homogenate was mixed with an equal volume of acid pepsin solution (5 mg/mL of 0.5 M acetic acid) and incubated overnight at 4 °C. Following centrifugation, the supernatant was assayed for soluble collagen content according to the manufacturer’s instructions. The absorbance at 555 nM was read on a VersaMax ELISA microplate reader (Molecular Devices, LLC., Silicon Valley, CA, USA). The lung collagen data were expressed as µg of soluble collagen per right lung of rat.

### 2.7. Assessment of Vascular Leakiness, Pulmonary Edema, and Profibrotic Cytokines

To determine lung vascular leakiness, the total protein content in BALF was measured using the bicinchoninic acid (BCA) assay. The absorbances were determined at 570 nM using the VersaMax ELISA microplate reader (Molecular Devices, LLC., San Jose, CA, USA). The protein amount was calculated based on a bovine serum albumin (BSA) standard curve and expressed in µg/mL of BALF. After BAL, the lungs were harvested, washed in 1X PBS to remove debris, blotted using tissue paper, and weighed (wet weight). The lung index was determined by dividing the wet lung weight with the body weight. Profibrotic/inflammatory cytokine levels in BALF harvested at predetermined time points after bleomycin challenge and control rats were analyzed using commercially available ELISA assays kits according to the manufacturer’s instructions. The level of each biomarker is expressed in terms of pg/mL of BALF. Quantikine ELISA kits for TGFβ1, TNFα, IL6, IL1β, CINC1, TIMP1, VEGF, and WISP1 were purchased from R and D system (Minneapolis, MN, USA).

### 2.8. Lung Homogenization and Western Blot Analysis

At the 7- and 14-day time points, the right lungs were collected in 1X PBS buffer containing protease and phosphatase inhibitors (Thermo Fischer Scientific, Waltham, MA, USA). The samples were homogenized and centrifuged for 10 min at 12,000 RPM at 4 °C. The supernatant was collected, and the protein concentration was determined using a BCA assay. Protein samples were separated on a Novex 4–12% Tris-glycine gel system (Invitrogen) and transferred overnight to nitrocellulose membranes (Novex). The membranes were blocked in superblock blocking buffer (Thermo Scientific) for 2 h at room temperature (RT) and incubated with primary antibodies in 0.1% TBST at 4 °C for 1 h or overnight. The primary antibodies included anti-P-smad2/3, -T-Smad, -galectin-3, -PDGF receptor-β, -phospho-AKT308/AKT, -procollagen1Aα, transglutaminase-2, -α-SMA, -vimentin, -GAPDH (Cell Signaling Technology, Danver, CO, USA), anti-TGFβ receptor-I, -fibronectin, and -CTGF (Abcam Inc., Waltham, MA, USA), whereas anti -LPA1, -LOXL2, and -procollagen 3Aα antibodies were purchased from Thermofisher Scientific, Rockford, USA. After washing with TBST, the membranes were incubated with horseradish peroxidase (HRP)-conjugated secondary (anti-rabbit or anti-goat) antibody (Abcam Inc., USA) for 1 h at RT at 1:10,000 dilution in 0.1% TBST. Immune complexes were detected using enhanced chemiluminescence (SuperSignal^®^ West Pico Chemiluminescent Substrate; Thermofisher Scientific, USA) by exposing them to X-ray film and developed using a SRX 101A, medical film processor (Konica Minolta; Medical and Graphics Inc., Changzhou, China). The signals were quantified using ImageJ software version 1.54.

### 2.9. Histopathological Evaluation of Pulmonary Fibrosis Progression

The left lungs were processed using a routine histology protocol. Paraffin-embedded tissue (4 μm slides) was stained with hematoxylin and eosin. Pathological changes in lung tissue were assessed using criteria adapted from a previously published protocol by Ashcroft et al. and Hubner et al. [38,39]. The criteria used for grading lung fibrosis were the following: 0: Alveolar septa: No fibrotic burden at the most flimsy small fibers in some alveolar walls, lung structure: Normal lung; 1: Alveolar septa: Isolated gentle fibrotic changes (septum ≤ 3× thicker than normal), lung structure: Alveoli partly enlarged and rarefied, but no fibrotic masses present; 2: Alveolar septa: Clearly fibrotic changes (septum > 3× thicker than normal) with knot-like formation but not connected to each other, lung structure: Alveoli partly enlarged and rarefied, but no fibrotic masses; 3: Alveolar septa: Contiguous fibrotic walls (septum > 3× thicker than normal) predominantly in whole microscopic field, lung structure: Alveoli partly enlarged and rarefied, but no fibrotic masses; 4: Alveolar septa: Variable, lung structure: Single fibrotic masses (≤10% of microscopic field); 5: Alveolar septa: Variable, lung structure: Confluent fibrotic masses (>10% and ≤50% of microscopic field), lung structure severely damaged but still preserved; 6: Alveolar septa: Variable, mostly not existent, lung structure: Large contiguous fibrotic masses (>50% of microscopic field), lung architecture mostly not preserved; 7: Alveolar septa: Non-existent, lung structure: Alveoli nearly obliterated with fibrous masses but still up to five air bubbles; and 8: Alveolar septa: Non-existent, lung structure: Microscopic field with complete obliteration with fibrotic masses. The severity of fibrotic changes in each lung section was assessed as a mean score of severity.

### 2.10. Statistical Analysis

All data are presented as the mean ± standard error of the mean (SEM). The data were analyzed using one-way ANOVA followed by Dunnett’s test for multiple comparisons or unpaired *t* test as applicable using GraphPad prism versions 9 and 10. A *p* value < 0.05 compared with control or day 0 was set as statistically significant.

## 3. Results

### 3.1. Bleomycin Concentrations and Phenotype of Experimental Lung Fibrosis in Rats on Day 14

**Figure 1:**
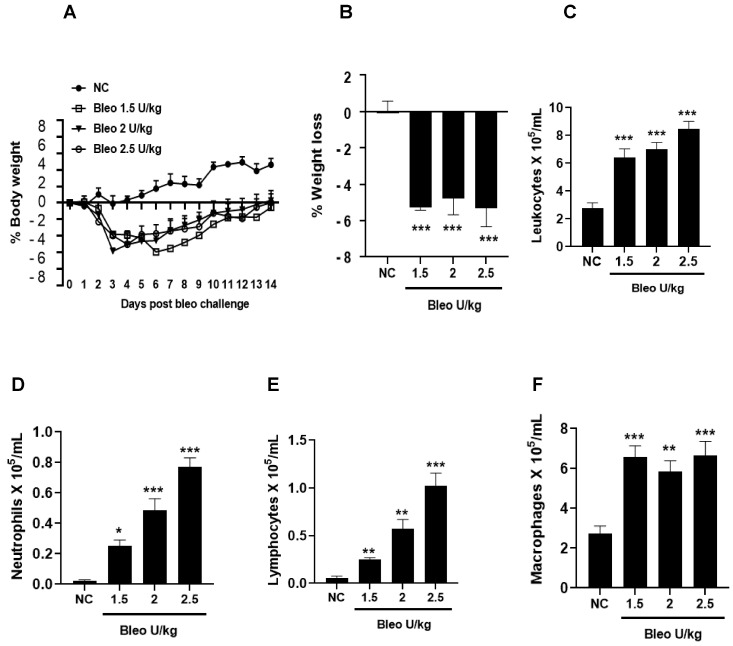
Effects of intratracheal (i.t) bleomycin concentrations on clinical signs and BALF pulmonary inflammation on day 14. Rats received the indicated amount of bleomycin i.t., as described in Materials and Methods. Body weight was measured daily, and inflammatory cells were counted after BAL on day 14. (**A**) % body weight, (**B**) % avg. body weight, (**C**) total leukocytes, (**D**) neutrophils, (**E**) lymphocytes, (**F**) macrophages. * *p* < 0.05; ** *p* < 0.01; and *** *p* < 0.001 vs. NC. *n* = 5 rats/group.

**Figure 2:**
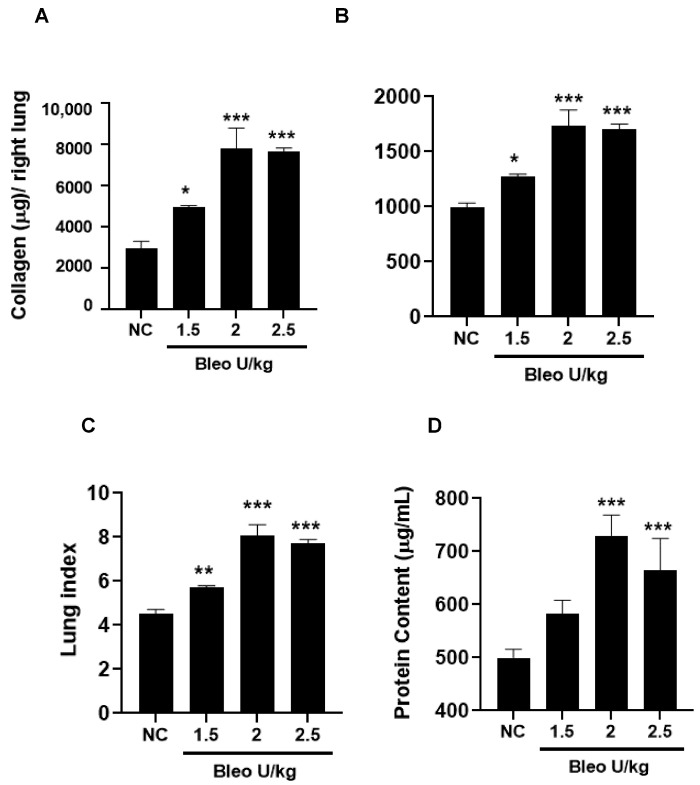
Effects of i.t bleomycin concentrations on lung parameters and vascular leakiness on day 14. Rats were treated with bleomycin, as described in Figure 1. After 14 days, lungs were harvested and BALF obtained. See Materials and Methods. (**A**) lung collagen content, (**B**) right lung (wet) weight, (**C**) lung index, (**D**) BALF protein content. * *p* < 0.05; ** *p* < 0.01; and *** *p* < 0.001 vs. NC. *n* = 5 rats/group.

**Figure 3:**
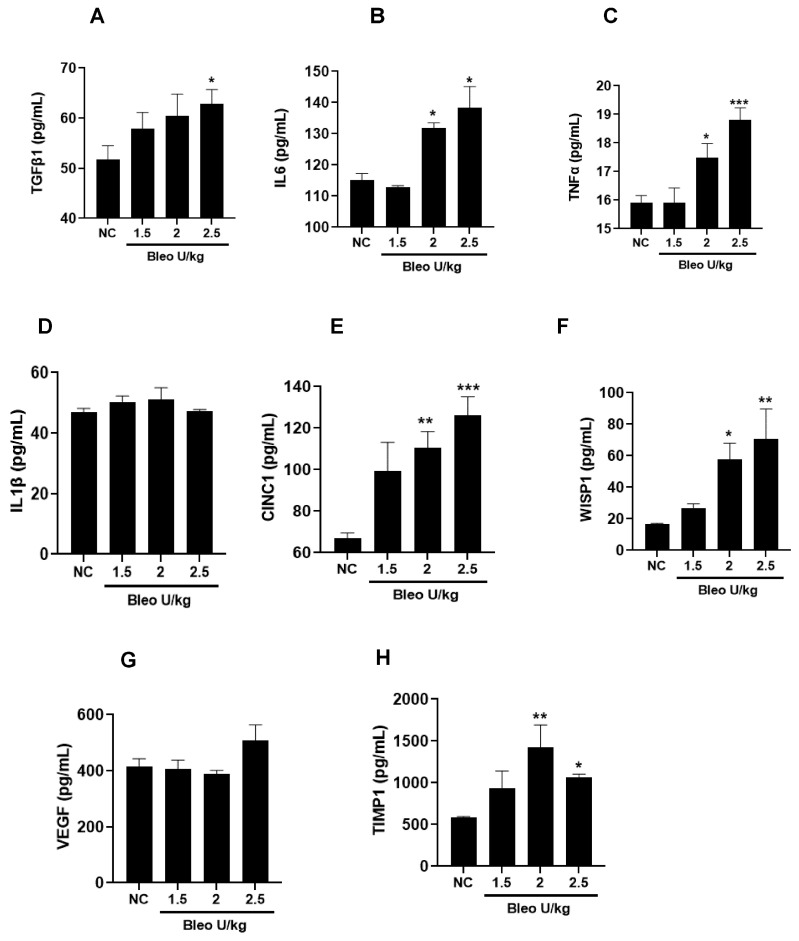
Effects of i.t bleomycin concentrations on BALF profibrotic/ fibrotic meditators on day 14. (**A**) TGFβ1, (**B**) IL6, (**C**) TNFα, (**D**) IL1β, (**E**) CINC1, (**F**) WISP1, (**G**) VEGF, (**H**) TIMP1. * *p* < 0.05; ** *p* < 0.01; and *** *p* < 0.001 vs. NC. *n* = 5 rats/group.

To test the effect of bleomycin (1.5, 2, 2.5 U/kg, i.t.) on pulmonary inflammation and selective biomarker concentrations in BALF on day 14, the rats were challenged with bleomycin and BAL after 14 days, as described in the Materials and Methods. The body weights of the rats were recorded daily until day 14. We observed similar and significant (*p* < 0.001) body weight loss without mortality at all bleomycin doses compared to NC (Figure 1A,B). The total leukocyte, neutrophil, lymphocyte, and macrophage counts in BALF significantly (*p* < 0.01) increased at all bleomycin doses compared to NC (Figure 1C–F). Similarly, the lung collagen content, lung weight and index, and BALF protein content increased significantly (*p* < 0.05) in all groups treated with bleomycin compared to NC (Figure 2A–D). The levels of BALF TGFβ1 were significantly (*p* < 0.05) increased in only the 2.5 U/kg bleomycin treatment. The levels of IL6, TNFα, CINC1, WISP1, and TIMP1 also showed significant increases (*p* < 0.05) with the 2 and 2.5 U/kg bleomycin treatments compared to controls. However, the levels of IL1β and VEGF remained unchanged at all bleomycin doses (Figure 3A–H).

### 3.2. Effects of Bleomycin on Clinical Signs on Days 3, 7, and 14

**Figure 4:**
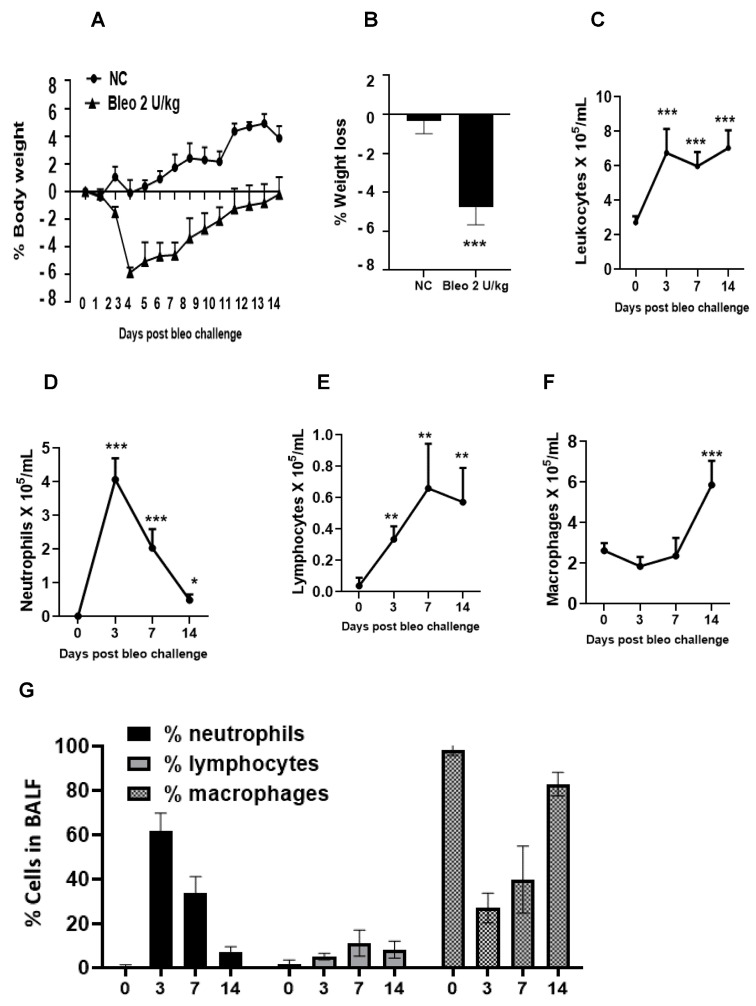
Effects of i.t. bleomycin on clinical signs and pulmonary inflammation on days 3, 7, and 14. (**A**) % body weight, (**B**) % avg. body weight loss, (**C**) total leukocytes, (**D**) neutrophils, (**E**) lymphocytes, (**F**) macrophages, (**G**) % BALF inflammatory cells. * *p* < 0.05; ** *p* < 0.01; and *** *p* < 0.001 vs. day 0. *n* = 5 rats/group.

Throughout the study, we monitored animals for clinical signs of body weight loss and mortality following the instillation of 2 U/kg i.t. bleomycin. We observed that the rats challenged with bleomycin showed a significant (*p* < 0.001) body weight loss compared to the controls. The maximum body weight loss was observed on day 4, which was sustained to a similar degree up to day 7. After that, there weight recovery was observed until the end of the study. No mortality (0%) was observed following the administration of 2 U/kg i.t. bleomycin until the end of the study (day 14) (Figure 4A,B). 

### 3.3. Kinetics of Pulmonary Inflammatory Cell Infiltration on Days 3, 7, and 14 Due to Bleomycin

We studied the inflammation caused by bleomycin by counting the inflammatory cells in BALF on days 3, 7, and 14 after administering 2 U/kg i.t. bleomycin. We observed a significant increase in total leukocyte counts at all three time points (Figure 4C). The number of neutrophils increased significantly (*p* < 0.01) on days 3, 7, and 14, with the highest increase observed on day 3 (Figure 4D). The lymphocyte counts also significantly (*p* < 0.01) increased at all time points (Figure 4E), while the macrophage counts increased significantly (*p* < 0.01) only on day 14 (Figure 4F) compared to day 0. The percentage of neutrophils in BALF was highest on day 3 at 60%, which decreased to 32% on day 7, and to less than 10% on day 14. The percentage of macrophages was highest on day 14 at 82%, whereas it was 40% on day 7 and 26% on day 3. Moreover, the percentage of lymphocytes remained at less than 10% throughout the time course. On day 0, BALF mainly consisted of macrophages, accounting for 100% of the cells (Figure 4G).

### 3.4. Kinetics of Lung Parameters and Vascular Leakiness on Days 3, 7, and 14 Due to Bleomycin

**Figure 5:**
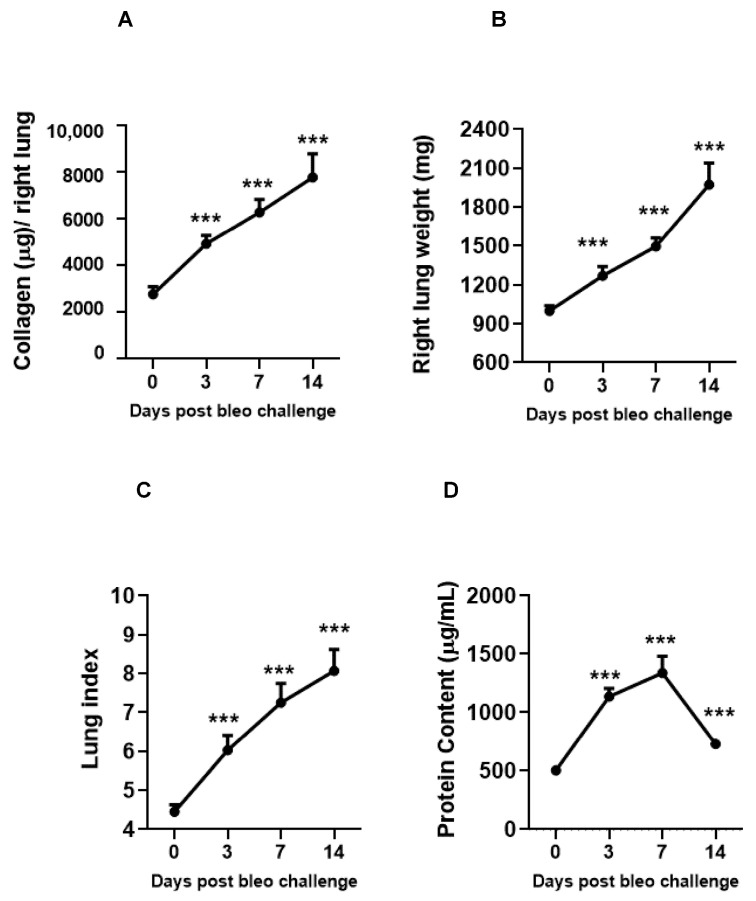
Effects of i.t. bleomycin on lung parameters and vascular leakiness on days 3, 7, and 14. (**A**) Lung collagen content, (**B**) right lung weight, (**C**) lung index, (**D**) BALF protein content. *** *p* < 0.001 vs. day 0. *n* = 5 rats/group.

The kinetics of fibrosis progression due to 2 U/kg i.t. bleomycin instillation were determined by assessing the lung collagen content, lung weight, and lung index. The vascular leakiness was estimated by BALF protein content on days 0, 3, 7, and 14. Bleomycin significantly (*p* < 0.01) and time-dependently (day 3 < day 7 < day 14) increased the collagen content, lung weight, lung index, and BALF protein content (day 14 < day 3 < day 7 compared to day 0) (Figure 5A–D).

### 3.5. Kinetics of Profibrotic Environment in the Lungs on Days 3, 7, and 14 Due to Bleomycin

**Figure 6:**
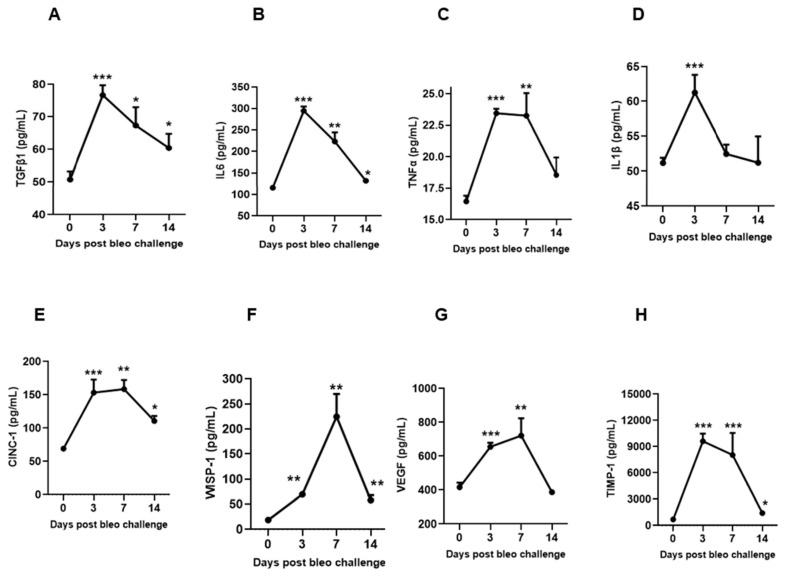
Effects of i.t. bleomycin on BALF profibrotic/fibrotic meditators on days 3, 7, and 14. Levels of BALF (**A**) TGFβ1, (**B**) IL 6, (**C**) TNFα, (**D**) IL1β, (**E**) CINC1, (**F**) WISP1, (**G**) VEGF, and (**H**) TIMP1. * *p* < 0.05; ** *p* < 0.01; and *** *p* < 0.001 vs. day 0. *n* = 5 rats/group.

To characterize the kinetic effects of 2 U/kg i.t. bleomycin on various biochemical markers of lung fibrosis, we measured the concentrations of TGFβ1, IL6, TNFα, IL1β, CINC1, WISP1, VEGF, and TIMP1 in BALF at days 3, 7, and 14 following instillation. On days 3 and 7, we observed a significant (*p* < 0.01) increase in the profibrotic mediators TGFβ1, IL6, TNFα, CINC1, WISP1, VEGF, and TIMP1, whereas a significant (*p* < 0.01) change in IL1β levels was seen only on day 3 in the bleomycin-challenged rats compared to day 0. On day 14, the levels of TGFβ, IL6, CINC1, WISP1, and TIMP1 remained significantly elevated compared to day 0. The levels of TGFβ1, IL6, and IL1β peaked on day 3, and then declined on days 7 and 14. The levels of TNFα, CINC1, VEGF, and TIMP1 also peaked on day 3, but stayed up to day 7; then, the levels declined towards day 14. The WISP1 levels increased on day 3, peaked on day 7, and further declined towards day 14 compared to day 0 (Figure 6A–H).

### 3.6. Bleomycin Activates Clinically Relevant Molecular Pathways and Protein Targets Linked to Fibrogenesis on Days 7 and 14

**Figure 7:**
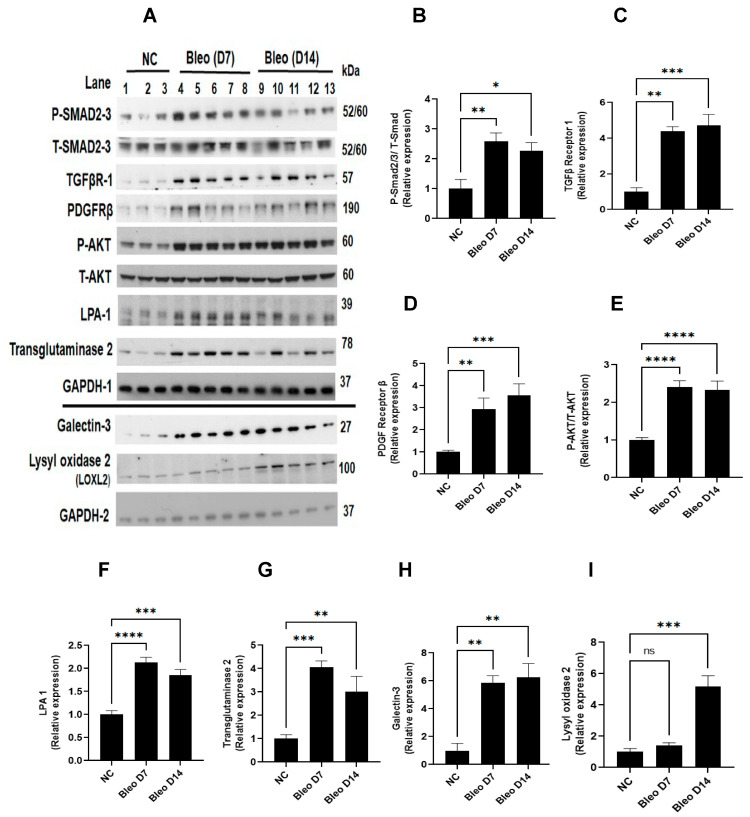
Effects of i.t. bleomycin challenge on TGFβ, PDGF/AKT pathways, and clinically relevant protein targets linked to fibrogenesis on days 7 and 14. Individual blots were performed for each protein and normalized with the average of 3 GAPDH blots. (**A**) Protein by Western blots; relative expressions of (**B**) P-Smad2/3, (**C**) TGFβ receptor 1, (**D**) PDGF receptor β, (**E**) P-AKT, (**F**) LPA-1, (**G**) transglutaminase-2, (**H**) galectin-3, and (**I**) lysyl oxidase 2. * *p* < 0.05; ** *p* < 0.01; *** *p* < 0.001, and **** *p* < 0.0001 vs. NC. *n* = 5 rats/group. Ns: Non significant.

To assess the involvement of molecular pathways (TGFβ, PDGF/AKT) and profibrotic mediators (galectin-3, LPA1, transglutaminase-2, and lysyl oxidase 2) during fibrogenesis on days 7 and 14, we studied the effect of 2 U/kg i.t. bleomycin on the protein expressions of these mediators (Figure 7A–I). Western blot analysis revealed a significant (*p* < 0.01) upregulation of P-Smad 2/3, TGFβ receptor- I, PDGF receptor -β, P-AKT -308, galectin-3, LPA-1, and transglutaminase-2 on days 7 and 14 compared to day 0. Lysyl oxidase -2 expression was upregulated only on day 14 (Figure 7A–I).

### 3.7. Bleomycin Increases the Expressions of Clinically Relevant EMT Biomarkers and ECM Components on Days 7 and 14

**Figure 8:**
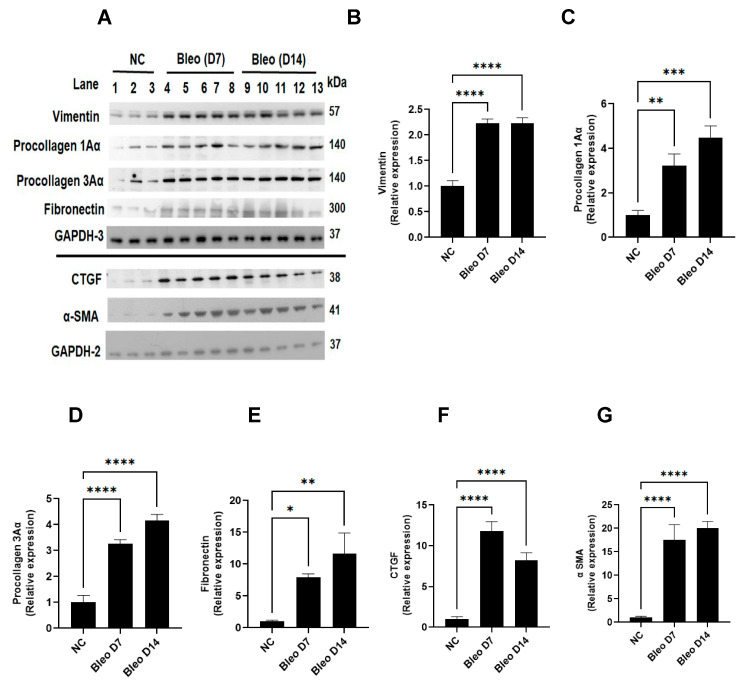
Effects of i.t. bleomycin challenge on the EMT process and ECM components on days 7 and 14. Individual blots were performed for each protein and normalized with the average of 3 GAPDH blots. (**A**) Protein by Western blots; relative expression of (**B**) vimentin, (**C**) procollagen-1Aα, (**D**) procollagen-3Aα, (**E**) fibronectin, (**F**) CTGF, and (**G**) α-SMA. * *p* < 0.05; ** *p* < 0.01; *** *p* < 0.001 and **** *p* < 0.0001 vs. NC. *n* = 5 rats/group.

We measured the effects of 2 U/kg i.t. bleomycin on the clinically important EMT process and ECM composition in fibrogenesis by quantifying vimentin, α-SMA, procollagen-1Aα, procollagen-3Aα, fibronectin, and CTGF in lung tissue, as described in Materials and Methods. Western blot analysis revealed a significant (*p* < 0.01) increase in vimentin, procollagen-1Aα, procollagen-3Aα, fibronectin, CTGF, and α-SMA expression on days 7 and 14 due to bleomycin (Figure 8A–G).

### 3.8. Bleomycin Induces Progressive Fibrotic Changes with Increased Severity from Days 3 to 7 and 14

**Figure 9:**
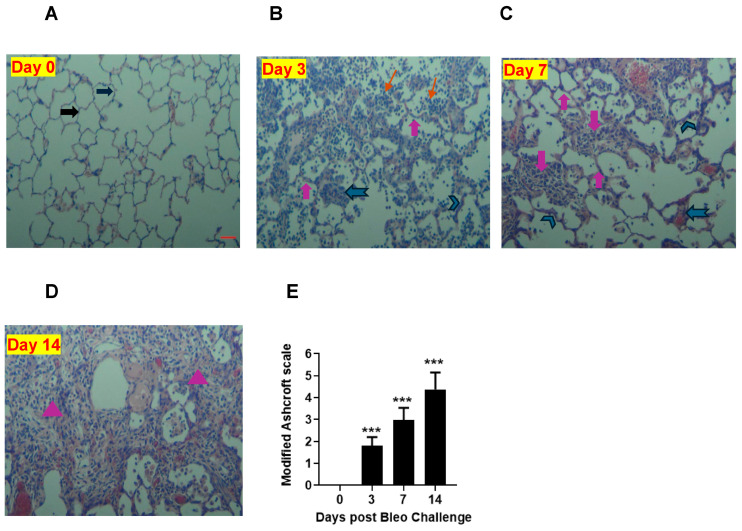
Effects of i.t. bleomycin challenge on lung histology and severity of fibrosis on days 3, 7, and 14. Representative histopathological images (10× magnification) and H&E lung staining are shown. (**A**) Normal rats (day 0). Rats treated with bleomycin and harvested on day 3 (**B**), day 7 (**C**), and day 14 (**D**). Severity of fibrosis post bleomycin by modified Ashcroft scale (**E**). Fibrosis was examined in upper, upper-mid, lower-mid, and lower lung sections (5 random fields per each). See Materials and Methods for details. Right block arrow: normal alveolar septa; upward block arrow: thickened alveolar septa; chevron arrow: contiguous fibrotic walls of alveolar septa; notched arrow to the left: single fibrotic masses; downward block arrow: confluent fibrotic masses; triangle: large contiguous fibrotic masses; line arrow: cellular infiltration. Data are expressed as mean ± SEM of *n* = 5 rats/group. *** *p* < 0.001 vs. day 0 using an unpaired *t*-test). Scale bar = 50 µm.

To quantify the severity of fibrosis, we conducted H&E staining to evaluate the pathological changes in lung sections on days 0, 3, 7, and 14. At day 0, the alveolar structure was normal, as shown in Figure 9. However, over time, we observed accumulations of leukocytes and thickening of the alveolar septa (days 3, 7, and 14). Additionally, we noticed the distortion of alveolar architecture and the deposition of fibrotic mass on days 7 and 14. The lung showed more fibrotic changes on day 14 than day 7 due to 2 U/kg i.t. bleomycin. We used a modified Ashcroft scale to quantify the severity of fibrosis. Our results revealed that the scores were significantly (*p* < 0.01) higher on days 3, 7, and 14 compared to the 0 time point (Figure 9A–E). 

## 4. Discussion

In the present study, we investigated the progression of lung fibrosis in the classical rat bleomycin model. We examined the development of fibrotic disease over time by studying clinical signs, lung histopathology, and relevant disease endpoints in the lung including pathways involved in inflammation, fibrogenesis, profibrotic mediator signaling, and biomarkers. Our findings showed that rats develop robust, progressive fibrotic disease in the lungs in a time-dependent manner that may differ from mice. Our study revealed that administering 2–2.5 U/kg of bleomycin led to an increase in inflammation and profibrotic mediators, ultimately resulting in the development of a fibrotic phenotype characterized by increased collagen without causing mortality. Knowing the appropriate doses of bleomycin that can cause appropriate disease phenotype without acute mortality can be very helpful for evaluating disease mechanisms and therapeutic outcomes.

Next, we investigated the kinetics of disease progression after bleomycin on days 0, 3, 7, and 14. When challenged with 2 U/kg i.t. bleomycin, the rats exhibited a progressive loss of body weight [40] until day 4 (maximum), after which they began to recover [41] as the study progressed. No mortality was observed. The total leukocytes count in BALF increased from day 3 until 14, suggesting continuous inflammation. The neutrophils peaked within 3 days and returned to base level by day 14 post bleomycin treatment. The total lymphocytes started increasing by day 3, peaked at day 7, and remained at the same levels until day 14. Macrophages peaked after 14 days, but no change was seen on days 3 and 7 after bleomycin treatment. Our data clearly show an inflammatory response attributed to neutrophils (day 3), lymphocytes, and macrophages (late) due to bleomycin [8]. The early increase in collagen in the lung on day 3 suggests the activation of fibrogenesis processes due to bleomycin, and further continuation on days 7 and 14 was confirmed by the increased collagen content (day 14 > day 7). During the experiment, we also observed an increase in lung weight and lung index from day 3 to 14. This suggests that rats developed a progressive fibrotic phenotype accompanied by an increased collagen content, lung weight, and lung index. The BALF protein content was greater on days 3 and 7 (peak) but decreased on day 14. Overall, the data suggest that the change in lung weight on day 14 seems to be attributed to lung collagen deposition, whereas on days 3 and 7, both edema and collagen production seem to be involved.

To investigate the time course profiles of mediators involved in fibrogenesis in the rat bleomycin model, the levels of TGFβ1, IL6, IL1β, TNFα, CINC1, WISP1, VEGF, and TIMP1 were examined in BALF. Proinflammatory cytokines IL6, IL1β, TNFα, and TGFβ1 are necessary for collagen production and fibrosis development [42,43,44,45,46]. Increased levels of IL6, IL1β, TNFα, and TGFβ1 have been detected in rodent bleomycin fibrosis models as well as IPF patients. Our study has confirmed that bleomycin induces greater production of BALF IL6, IL1β, TNFα, and TGFβ1 on days 3 and 7 compared to day14. We confirmed their expressions and presumed functions in the early and mid-stages of disease progression. Along with profibrotic cytokines, CINC1, a potent neutrophil chemoattractant, is involved in disease progression [22]. Our study showed that BALF CINC1 levels increased and peaked on day 3, and sustained until day 7 before declining on day 14. Our data also suggest a major role of neutrophils along with other profibrotic mediators during progressive fibrosis. Increased expression of WISP1 is reported in type II AECs in fibrotic lungs of rodents and IPF patients [47]. WISP1 is involved in impaired epithelial–mesenchymal crosstalk in pulmonary fibrosis, induces IL6 expression, and promotes pro-proliferative effects on fibroblasts [48]. Our study found that WISP1 in BALF increased progressively from day 3 to day 7 (peak), and then declined on day 14. VEGF, which has mitogenic and profibrotic effects on fibroblasts, has been implicated in the pathogenesis of IPF [49]. We showed that VEGF was upregulated on days 3 and 7 but not day 14. Early altered regulation of TIMP1 following bleomycin administration has been reported in bleomycin-induced pulmonary fibrosis [50]. In our study, TIMP1 increased on day 3, was sustained up to day 7, and declined on day 14. Our kinetics data suggest a specific peak pattern of profibrotic mediators. The parallel early increase (day 3) of profibrotic cytokines and collagen suggests their contribution, and confirms their role in early fibrogenesis and collagen production. Furthermore, our data also suggest that the profibrotic cytokines not only initiate the process of fibrogenesis, but that their involvement continues up to day 7 to the same or lesser extent as on day 3. Their decreased levels on day 14 suggest that their active role and involvement decline toward day 14. This also suggests that other mediators, target proteins, and fibrotic principal pathways take over the collagen production to keep the fibrogenesis process active and increase the severity of fibrosis in rat lungs. 

To investigate the time course profiles of clinically relevant pathways and molecular targets involved in fibrogenesis in the rat bleomycin model, we also examined the expressions of the TGFβ1/Smad pathway, PDGF/AKT pathway, galectin-3, transglutaminase 2, LPA-1, and LOXL-2 proteins in lung homogenates harvested on days 7 and 14. TGFβ is a major profibrotic cytokine that increases the transcription of target genes, such as procollagen I and III, via transmembrane receptor serine/threonine kinases and the cytoplasmic Smad-2/3 signaling pathways [51]. In this study, we showed increased expressions of p-Smad2/3 and TGFβ receptor I proteins at days 7 and 14, which is indicative of an activated TGFβ-Smad-dependent pathway. Abnormal expression of PDGF has been linked to the development of pulmonary fibrosis. PDGF-A and PDGF-B mRNA increased in BALF from lungs of bleomycin-treated hamsters [52] and PDGFR-β-dependent pathways appear to strongly contribute to the progression of pulmonary fibrosis [53]. The role of AKT has been reported in disease onset and progression in pulmonary fibrosis by promoting myofibroblast differentiation and ECM deposition [54]. These reports suggest PDGF/AKT pathway axis contribution in fibrosis development. We showed increased expressions of PDGFR-β and P-AKT following bleomycin instillation on days 7 and 14, suggesting an activated PDGF/AKT axis during disease progression in rats. Galectin-3 is increased in the BALF of IPF patients and in rodent bleomycin models [28,34], and is implicated in EMT [55]. Studies demonstrated that LPA-1 levels are elevated in BALF due to bleomycin [56], and promote epithelial cell apoptosis as well as fibroblast recruitment and vascular leaking [57]. Transglutaminase 2 is known to induce a myofibroblast phenotype, and plays an important role in pulmonary fibrogenesis. It could potentially represent an interesting therapy target for IPF [35,58]. We showed that galectin-3, transglutaminase -2, and LPA-1 expression strongly increased on days 7 and 14 during disease progression. The final step in ECM maturation is cross-linking of collagen and elastin via oxidation of lysyl amine residues. This process is mediated by members of the lysyl oxidase family [59,60]. LOXL2 proteins are detected in activated fibroblasts, reactive pneumocytes, and the vasculature in fibrotic foci of fibrotic lung tissue of IPF patients [61]. LOX primarily acts as a critical regulator of the inflammatory response and subsequent fibrosis process after lung injury [62]. In our study, increased expression of LOXL2 on day 14 (late) suggests that cross-linking of elastin and collagen fibrils in ECM begins after day 7 and continues until day 14 in the rat model.

Lastly, to validate and correlate the role of key drivers (mechanistic readouts) in the development of fibrosis, we used standard measures such as EMT, ECM components, and histopathology. Vimentin, β-catenin, and α-SMA are mesenchymal biomarkers whose expression is aberrantly increased as EMT progresses [63]. We report increased protein expressions of vimentin and α-SMA on days 7 and 14, suggesting myofibroblast activation, which plays an important role in ECM production during the proliferative and remodeling phases [64]. Collagen is a pivotal ECM component [65], and procollagen 1Aα- and 3Aα-dependent cellular processes are implicated in IPF [66], together with an aberrant CTGF increase, a key step in the formation of new ECM, and the assembly of a fibrillar fibronectin matrix, which serves as a scaffold for the binding of collagens and other ECM proteins [67]. In our study, both procollagen 1Aα and 3Aα as well as CTGF and fibronectin protein expression were elevated on days 7 and 14, suggesting that the ECM remodeling correlates with the increase in collagen on days 7 and 14, culminating in the deposition of fibrotic mass within lung tissue. We observed variable degrees of fibrotic changes (heterogenous) due to bleomycin. In the bleomycin-treated rats, there was more accumulation of leukocytes and thickening of alveolar septa on day 3, whereas more distortion of alveolar architecture with clearly more fibrotic mass was noted in the lung on day 14 than on day 7; this correlates with the severity of fibrosis quantified using modified Ashcroft scores. This suggests a progressive fibrotic phenotype in the rats after a single i.t. bleomycin challenge, which is consistent with collagen readout.

We have provided critical information on the contributions of immune cells as well as on selected profibrotic cytokines/chemokines during disease progression in three distinct phases in the bleomycin rat model, which could serve as the base for future immunopharmacological interventions. Our data of pulmonary cell infiltration analysis revealed that the rat model exhibited a shorter inflammatory phase than expected from mouse studies of approximately 3 days post bleomycin; this is supported by detectable fibrogenesis on day 3 along with a profound increase in profibrotic soluble mediators within BALF. This difference was not expected, and may indicate that the rat model is a better alternative than the mouse model. 

Targeting specific profibrotic immune cell or profibrotic cytokines/chemokines by monoclonal antibodies or small molecules may pave the way for novel pharmacological interventions for treating pulmonary fibrosis [68]. Our data suggest days 3 and 7 are good time points for specific drug treatments. Recently, inhibitors of TGFβ (1D11) [69], PDGF (Nintedanib) [20], AKT (triciribine) [42], galectin-3 (TD139) [70], LPA-1 (AM966) [40], and LOXL2 (AB0023) [56] have shown promising and beneficial effects in rodent models of lung fibrosis. Furthermore, a humanized TGFβ-neutralizing antibody (Fresolimumab) and novel inhibitors of galectin-3, LPA1, LOXL2, PDGF, and AKT are being tested in clinical trials for potential IPF treatment. In our study, we studied the expression kinetics of these clinically relevant principal target proteins, showed activated TGFβ1-Smad dependent and PDGF/AKT pathway axes as well as increases in galectin-3, transglutaminase 2, LPA1-1, and LOXL2 proteins due to bleomycin, which is consistent with previous reports [26,27,28,29,30,31,32,33,34,35,36,51,52,53,54,55,56,57,58,59,60,61,62,70]. The framework of endpoints analyzed in this study has translational value and is meaningful for therapeutic interventions. This knowledge may be helpful for determining appropriate experimental designs as well as endpoint selection for phenotype and efficacy assessment. The early evaluation of therapeutics on days 3 and 7 may expedite the validation and proof of principle of novel targeted therapeutics. Further validation can be obtained by extending the evaluation to day 14. In this study, we optimized the collagen content assay in the rat lung, which is the primary endpoint of fibrotic disease efficacy evaluation. This assay is the deciding factor for the selection and further development of the therapeutic under investigation. In this research, we are highlighting a model without mortality and acceptable weight loss, suggesting optimized conditions to use the model successfully to explore possible novel mechanisms and therapeutic targets of IPF. These data, which have translational value, can be utilized to study the mechanisms of potential antifibrotic novel treatments. This is a valuable tool, as it helps in selecting the most promising candidate for successful translation into clinical trials. 

Our study has certain limitations. We evaluated only a few target proteins and pathways, without measuring lung function. Therefore, additional efforts are necessary to understand the complex progression of the disease. Using Masson’s trichrome staining could be helpful to increase the accuracy of determining the percentage of fibrotic lung tissue over time. Our findings could be further expanded using flow cytometry and immunophenotyping. Next-generation sequencing, particularly single-cell sequencing, could be used to gain novel insights into fibrosis disease stages in this rat model. Our framework of translational readouts along with lung function measurements could further strengthen mechanism studies of novel antifibrotic therapies.

## 5. Conclusions

This study provides significant insights into the early, mid, and active stages of fibrosis in rats by quantifying recognized outcome measures in the classical rat bleomycin model. It provides valuable data on the kinetics of key drivers involved in the progressive development of lung fibrosis in rats. Additionally, our framework of translational readouts, along with lung function measures using suitable imaging modalities, could add value to the translational success. These insights will be useful in the preclinical development of potential novel single or combination therapies, as well as in exploring their mechanisms of action.

## Data Availability

All relevant data are within the manuscript. Further inquiries can be directed to the corresponding author.
